# Sclerophyllous structural and chemical traits of mite‐induced galls on *Crinodendron patagua* (Elaeocarpaceae) leaves

**DOI:** 10.1111/plb.70246

**Published:** 2026-06-19

**Authors:** L. M. Guedes, L. Rodríguez‐Cerda, N. Aguilera, E. Grandón, F. R. Valeriano, J. F. de Lima, J. F. P. de Oliveira, B. G. Ferreira

**Affiliations:** ^1^ Laboratorio de Semioquímica Aplicada, Facultad de Ciencias Forestales Universidad de Concepción Concepción Chile; ^2^ Departamento de Botânica, Instituto de Biologia Universidade Federal do Rio de Janeiro Rio de Janeiro Brazil; ^3^ Programa de Pós‐graduação em Ecologia, Conservação e Biodiversidade, Instituto de Biologia Universidade Federal de Uberlândia Uberlândia Brazil

**Keywords:** Cell walls, drought stress, Eriophyidae, gall‐inducing mites, Mediterranean‐type ecosystems

## Abstract

- Sclerophyllous plants present a suite of traits, including small, leathery leaves with thick cuticles, dense trichomes and sclerified tissues, which are often accompanied by high levels of secondary metabolites. Although these features are generally associated with protection and defence, they do not necessarily deter gall‐inducing organisms. In fact, some gall inducers may exploit the structural and chemical complexity of sclerophyllous tissues to develop protective and nutrient‐rich microhabitats. This appears to be the case for eriophyid mites (Acari: Eriophyidae) that induce galls on the leaves of *Crinodendron patagua* Mol. (Elaeocarpaceae), a sclerophyllous species endemic to Chile and characteristic of coastal Mediterranean forests. We hypothesized that these mites exploit and enhance the morphoanatomical and chemical traits of *C. patagua* sclerophyllous leaves to induce the formation of galls that function as protective, nutrient‐enriched microhabitats in the stressful Mediterranean‐type climate of central Chile.
- Anatomical, cytohistometric, histochemical and spectrophotometric analyses were performed on galled and non‐galled leaves to evaluate changes in anatomical structure, primary metabolites (proteins and reducing sugars) and secondary metabolites (polyphenols).
- The results revealed that mites induce anatomical changes that favour progeny development, including hypotrophy and thinning of adaxial epidermal cell walls rich in cellulose and hemicellulose and the accumulation of proteins and reducing sugars in these nutritive cells. In parallel, lignified trichomes redifferentiated at the opening of the gall, abaxial epidermal cells exhibited hypertrophy and thickened walls, and the polyphenol content increased in gall tissues. Some sclerophyllous characteristics were intensified, whereas others remained unchanged.
- Gall development in *C. patagua* selectively reprograms structural and biochemical traits to meet the nutritional and protective needs of eriophyid mites, partially supporting our original hypothesis.

## Graphical abstract

Eriophyid mites reprogram the structural and biochemical traits of sclerophyllous leaves in *Crinodendron patagua*, promoting the formation of nutrient‐rich and protective gall tissues.

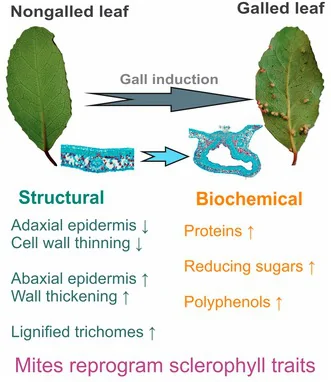

## INTRODUCTION

Sclerophyllous plants exhibit a suite of traits, including small, coriaceous leaves with thick cuticles, dense trichomes and high tissue rigidity, which are often associated with high concentrations of secondary metabolites such as alkaloids and phenolics (Medina *et al*. 1990; Hanley *et al*. 2007; Heras *et al*. 2020). These traits are generally regarded as the outcome of chronic environmental stress, such as seasonal drought, high irradiance and nutrient limitation, and, in turn, may contribute to water conservation and the deterrence of herbivory (Turner 1994; Hanley *et al*. 2007; He 2021). However, evidence suggests that such characteristics may not deter gall‐inducing organisms, which are remarkably rich in distribution in these environments (Price *et al*. 1998; Espírito‐Santo & Fernandes 2009). Indeed, studies on systems such as *Paleomystella oligophaga* Becker & Adamski, 2008 (Lepidoptera) have reported galls on *Macairea radula* DC. (Melastomataceae) (Rezende *et al*. 2019, 2020) and on superhost species such as *Copaifera langsdorffii* Des. (Fabaceae) (Fagundes *et al*. 2020; Santos‐Silva & Araújo 2020) demonstrated that the accumulation of phenolic derivatives and stress‐related structural traits does not necessarily prevent gall establishment.

Furthermore, sclerophyllous traits may provide structural and chemical advantages for gall development (Price *et al*. 1998; Ribeiro & Basset 2007; Ribeiro *et al*. 2016; Csóka *et al*. 2017). Gall‐inducing species can exploit sclerophyllous tissues, creating a protected microenvironment within the gall (Ribeiro & Basset 2007; Ribeiro *et al*. 2014). However, experimental evidence demonstrating how specific structural and chemical traits of galls confer protection or nutritional benefits remains scarce and is largely restricted to a few systems (*e.g*., Rezende *et al*. 2020). Anatomical traits such as increased leaf thickness, dense indumentum and reinforced epidermal and mesophyll layers, along with the localized accumulation of secondary metabolites (*e.g*., alkaloids, terpenoids and phenolics), are frequently observed in gall tissues and have been proposed as traits that may contribute to gall function under stressful conditions (Ferreira *et al*. 2019; Kuster *et al*. 2019; Rezende *et al*. 2020; Guedes *et al*. 2024; Isaias *et al*. 2024; Fernandes *et al*. 2025).

The Mediterranean‐type ecosystems of central Chile, characterized by dry summers and wet winters, are among the five Mediterranean‐type ecosystems that occur globally (Bambach *et al*. 2013). Sclerophyllous forests in south‐central Chile (32°–40° S) host diverse evergreen species (Venegas‐González *et al*. 2023) that exhibit classic sclerophyllous traits: thick, leathery leaves with reinforced mesophyll, abundant sclerenchyma and very thick cuticles (Read *et al*. 2016). While these traits are metabolically costly, they provide advantages in nutrient‐poor soils and under herbivore pressure (Sánchez‐Gómez *et al*. 2008).

In addition to their high floristic diversity, Chilean sclerophyllous forests harbour largely unexplored fauna of gall‐inducing organisms (Quintero *et al*. 2014). These organisms are capable of overcoming the anatomical and chemical barriers typical of sclerophyllous plants; in doing so, they may intensify local tissue stress, potentially enhancing sclerophyllous traits in galls, which could confer advantages to gall inducers, although this hypothesis remains to be tested. *Crinodendron patagua* Mol. (Elaeocarpaceae), a sclerophyllous species endemic to Chile and emblematic of coastal Mediterranean forests, represents a suitable system to explore this possibility, as it hosts leaf galls induced by eriophyid mites (Nuñez & Saiz 1994). Although the gall inducer has not yet been formally described, Nuñez & Saiz (1994) identified the inducing organism as a member of the family Eriophyidae (Acari).

*Crinodendron patagua* occurs from Valparaíso to the Biobío region and typically grows in moist microhabitats such as streambanks, ravine bottoms and other shaded, humid environments (Rodriguez *et al*. 2018). This species is currently listed as vulnerable because of habitat loss, prolonged drought and overextraction of water for agriculture and industry, leading to significant population decline and degradation of its habitat. Its leaves are simple, coriaceous and arranged oppositely; they possess unicellular trichomes and anomocytic stomata restricted to the abaxial surface (Barrera & Meza 1997). Although the chemical composition of *C. patagua* remains unexplored, other Elaeocarpaceae species—such as *C. hookerianum* Gay and *Aristotelia chilensis* (Molina) Stuntz—have been reported to contain phenolic compounds and alkaloids in their fruits and leaves (Bittner *et al*. 1973; Quevedo *et al*. 1981; Céspedes *et al*. 2010; Adsersen *et al*. 2013; Rodríguez *et al*. 2021). These findings suggest that *C. patagua* likely shares similar chemical defence mechanisms.

On the abaxial surface of *C. patagua* veins, eriophyid mites (Acari: Eriophyoidea) induce globoid galls (Nuñez & Saiz 1994). In other mite‐induced gall systems, gall formation involves extensive histological modifications and coordinated transcriptomic and metabolic reprogramming of host tissues (Paponova *et al*. 2018; Guedes *et al*. 2022, 2024; Yang *et al*. 2023; Morina *et al*. 2025). However, how eriophyid mites manipulate the anatomical and biochemical traits of sclerophyllous *C. patagua* leaves to induce gall formation under Mediterranean‐type environmental stress remains unclear. We hypothesize that eriophyid mites exploit and enhance the morphoanatomical and chemical traits of the sclerophyllous leaves of *C. patagua* to induce the formation of galls that function as protective, nutrient‐enriched microhabitats in the stressful Mediterranean‐type climate of central Chile. To address this hypothesis, we aimed (i) to compare the morphoanatomical structure of galled and non‐galled leaves of *C. patagua*, focusing on traits associated with sclerophylly and gall structure, and (ii) to quantify and compare the concentrations of primary metabolites (*e.g*., sugars, starch, proteins) and secondary metabolites (*e.g*., phenolics, flavonoids) between galled and non‐galled tissues.

## MATERIALS AND METHODS

### Study area

The study area is located on the Concepción Campus of the Universidad de Concepción, Biobío Región, Chile (36° 49.655′ S; 73° 2.124′ W) (Supporting Information S1). This area is a remnant of a plain at the foot of Cerro Caracol, with humid soils and approximately 22% of the plant species present are native. The population of *C. patagua* (Supporting Information S1) is mixed with those of other native tree species, such as *Luma apiculata* (DC.) Burret (Myrtaceae), *Peumus boldus* Molina (Monimiaceae), *Quillaja saponaria* Molina (Quillajaceae) and *Maytenus boaria* Molina (Celastraceae), among others, as documented in the institutional flora records (Universidad de Concepción 2021).

The climate of Concepción city is temperate Mediterranean with oceanic influence (Csb), characterized by a rainy autumn–winter season and a dry spring–summer period (Table 1). Climatic data for 2024 are presented to characterize the environmental conditions of the study area. The sampling was conducted in August, which was during the winter season; June had the highest precipitation, and January was the driest month (Table 1). The relative humidity ranged from approximately 68–71% in summer to more than 85% in winter (Table 1).

**Table 1:** Climatic variables recorded in the city of Concepción, Biobío Region, Chile, from January to August 2024.

months	average temperature (°C)	maximum temperature (°C)	minimum temperature (°C)	relative humidity (%)	precipitation (mm)
January	17.4	**23.2**	12.5	**68.0**	**0**
February	**17.6**	**23.2**	**13.2**	71.0	0.2
March	15.7	21.3	10.7	71.7	35.4
April	13.0	17.5	9.3	81.3	77.2
May	9.2	13.7	5.3	83.0	99.2
June	10.9	13.7	8.4	**86.3**	**397.6**
July	**7.7**	**12.8**	**3.2**	78.0	62.8
August	9.0	13.5	5.1	80.7	113.0

The maximum and minimum values for each variable are highlighted in bold. Data were obtained from the Carriel Sur Meteorological Station (ID: 360019), provided by the Dirección Meteorológica de Chile, Dirección General de Aeronáutica Civil (https://climatologia.meteochile.gob.cl/, accessed July 2025).

### Plant material

Five *C. patagua* trees (n = 5) were selected, each approximately 8 m in height (Supporting Information S1). Leaves with and without galls were collected from lateral branches at a height of approximately 2 m (Supporting Information S1) in August 2024 at the end of the winter. Sampling was consistently performed on branches oriented towards the east (sunrise‐facing side) to minimize variation associated with cardinal orientation. The collected leaves with and without galls were one‐year‐old and were selected between the third and seventh nodes. Galls were collected during the maturation stage, with a diameter ranging from 1.0 to 1.9 mm. The number of leaves collected was adjusted to meet the requirements of each study.

### Leaf area and dry mass per area

For biomass and leaf area measurements, a total of 24 galled and 24 non‐galled leaves were collected from four individual trees (*n* = 6 leaves per category per tree). To ensure high comparability and minimize environmental variation, the sampling was restricted to the second and third nodes on east‐facing branches at a height of 2 m. The number of sampled trees was reduced to four because one of the selected trees did not present non‐galled leaves under the specified sampling criteria. The non‐galled and galled leaves were scanned in a scanner (HP Deskjet 1515, USA) with an associated scale. The leaf area was measured, and the number of galls per leaf was determined from the images with ImageJ software. After scanning, the leaves were oven‐dried (MMM Venticell 111 ECO, Germany) at 40 °C and weighed to determine their dry mass. The leaf mass per area, a commonly used index of sclerophylly (Groom & Lamont 1999), was then calculated using the following equation:

$$\text{Leaf mass}\;\mathrm{per}\;\text{area}=\frac{\mathrm{Dry}\;\text{mass}\;\left(g\right)}{\text{Leaf area}\;\left({\mathrm{mm}}^{2}\right)}$$

### Anatomical characterization of non‐galled and galled leaves

Anatomical descriptions of the non‐galled leaves and galls of *C. patagua* were obtained from samples collected from the sixth and seventh nodes of five different trees (n = 5 per organ). In galled leaves, only the gall tissues were analysed. Leaves were sectioned at their media third and fixed either in formalin–glacial acetic acid‐70% ethanol (Johansen 1940) for light microscopy or in 2.5% glutaraldehyde (Coetzee & van der Merwe 1984) for electron microscopy studies.

For light microscopy, the samples were dehydrated, embedded in Paraplast®, sectioned at 12 μm, deparaffinized, and stained with 0.5% astra blue and 0.5% safranin (9:1; v:v) (Kraus & Arduin 1997). Additional fragments of non‐galled and galled leaves (n = 5 for each category) were dehydrated, embedded in historesin, sectioned at 5–8 μm and stained with toluidine blue O (0.05%) (O'Brien & McCully 1987) in acetate buffer (pH 4.7). Anatomical preparations were documented using a Leica DM 500 light microscope equipped with a digital camera. For scanning electron microscopy (SEM), samples of non‐galled and galled leaves (n = 5 per condition) were dehydrated, mounted on aluminium stubs using double‐sided carbon tape, sputter‐coated with gold and examined with a Thermo Fisher Quattro S scanning electron microscope. Additional details on the microscopy preparation and imaging procedures are provided in Supporting Information S2.

### Cytohistometric measurements

Cytohistometric measurements were carried out on the photomicrographs obtained for anatomical analysis using ImageJ software. Images of non‐galled and galled leaves (n = 5 per condition) were analysed, with three sections selected from each sample (resulting in 15 measurements per variable). The measured parameters in the non‐galled leaves included the thickness (μm) of the adaxial and abaxial epidermis, mesophyll thickness, thickness of the adaxial and abaxial epidermal cell walls, and area (μm^2^) of the adaxial and abaxial epidermal cells. In galls, the corresponding parameters were the thickness of the inner epidermis (bordering the gall chamber) and outer epidermis, the gall cortex thickness, the thickness of the inner and outer epidermal cell walls, and the area of the inner and outer epidermal cells.

### Histolocalization of primary and secondary metabolites

Fresh galled and non‐galled leaves were collected from the sixth and seventh nodes of five different trees (n = 5 per organ), and freehand sections were prepared for the histochemical detection of reducing sugars, starch, proteins and lignin. For the detection of soluble phenols and flavanols, leaf fragments (n = 5) were previously fixed in formalin–ferrous sulphate and caffeine–sodium benzoate solutions, respectively. The protocols utilized for these histochemical analyses, including reagents and references, are summarized in Table 2, while specific methodological details and reaction colours are provided in Supporting Information S2. All reactions were monitored and photographed under a Leica DM 500 light microscope equipped with a digital camera. Stained sections were compared against unstained sections (blank) of both organs.

**Table 2:** Histochemical methods and reagents used for the detection of metabolites in *Crinodendron patagua* leaves.

target metabolites	method or reagent	references
Reducing sugars	Fehling's reagent	Sass (1951)
Starch	Lugol's reagent	Johansen (1940)
Proteins	Bromophenol blue	Baker (1958)
Total Phenols	Ferrous sulphate–formalin	Johansen (1940)
Flavonols	DMACA solution	Feucht *et al*. (1986)
Lignin	Acidified phloroglucinol	Johansen (1940)

### Biochemical analysis

All colorimetric measurements were carried out using a microplate reader (ELX800, Bio‐Tek), and the absorbance of each sample was measured in triplicate. The detailed procedures for all the biochemical analyses are provided in Supporting Information S2.

#### Quantification of soluble sugars and starch in leaf tissue

Total soluble sugars (TSS) and starch were quantified from 15 mg of leaf tissue collected from four different trees (n = 4), including both galled and non‐galled leaves of *C. patagua*. Leaf samples collected from the second and third nodes, previously used for dry mass determination, were homogenized and the TSS were extracted using a methanol:chloroform:water mixture following established protocols (Rose *et al*. 1991), with phase separation as described by Dickson (1979). Starch was subsequently recovered from the remaining pellet and enzymatically solubilized with amyloglucosidase. Both the TSS and starch contents were quantified colorimetrically using the phenol–sulphuric acid method (Chow & Landhäusser 2004). Concentrations were calculated from sucrose and glucose standard curves, respectively, and expressed as milligrams per gram of dry weight (mg g^−1^ DW).

#### Quantification of soluble proteins

The soluble protein content was quantified in fresh non‐galled and galled leaves (n = 4 per category) collected from the third and fourth nodes, following the Bradford assay (Bradford 1976). Protein concentrations were determined using a bovine serum albumin (BSA) standard curve and expressed as milligrams of protein per gram of fresh weight (mg g^−1^ FW).

#### Quantification of polyphenols and flavonoids

Methanolic extracts (0.1 g mL^−1^) obtained from a portion of the leaf samples previously collected from the second and third nodes for dry mass determination (n = 4 trees), including both galled and non‐galled leaves, were used to quantify total polyphenols and flavonoids. The total polyphenol content was determined using the Folin–Ciocalteu method (Singh *et al*. 2016), whereas the total flavonoid content was quantified using the aluminium chloride colorimetric assay (Kim 2003). The results were calculated from gallic acid and quercetin standard curves and expressed as milligrams of gallic acid equivalents per gram of dry weight (mg GAE g^−1^ DW) and milligrams of quercetin equivalents per gram of dry weight (mg QE g^−1^ DW), respectively.

### Data analysis

Statistical analyses were conducted to evaluate differences between galled and non‐galled leaves across morphological, cytohistometric and biochemical variables. Linear mixed‐effects models (LMMs, with the lme4 package) were used to assess differences in leaf area, adaxial epidermis thickness, mesophyll thickness, abaxial epidermis thickness and the area of adaxial epidermal cells. Generalized linear mixed‐effects models (GLMMs) fitted with a gamma distribution were used to analyse the variation in leaf dry mass (with a log link function), the thickness of the cell walls in the adaxial and abaxial epidermis, and the area of abaxial epidermal cells (also with a log link function). In addition, the contents of phenolic compounds, flavonoids, starch, proteins and sugars were evaluated using GLMMs with a gamma distribution, with an identity link function applied to the sugar content model. In all the models, the plant individual was included as a random effect to account for within‐plant variability, whereas the leaf type (galled *versus* non‐galled) was included as a fixed effect. Model adequacy was verified through residual normality and homoscedasticity assessments with the DHARMa package. All the statistical analyses were performed using R version 4.5.0 (R Development Core Team 2024), and the significance level was 5%.

## RESULTS

### Morphological and morphometric characterization

**Fig. 1:**
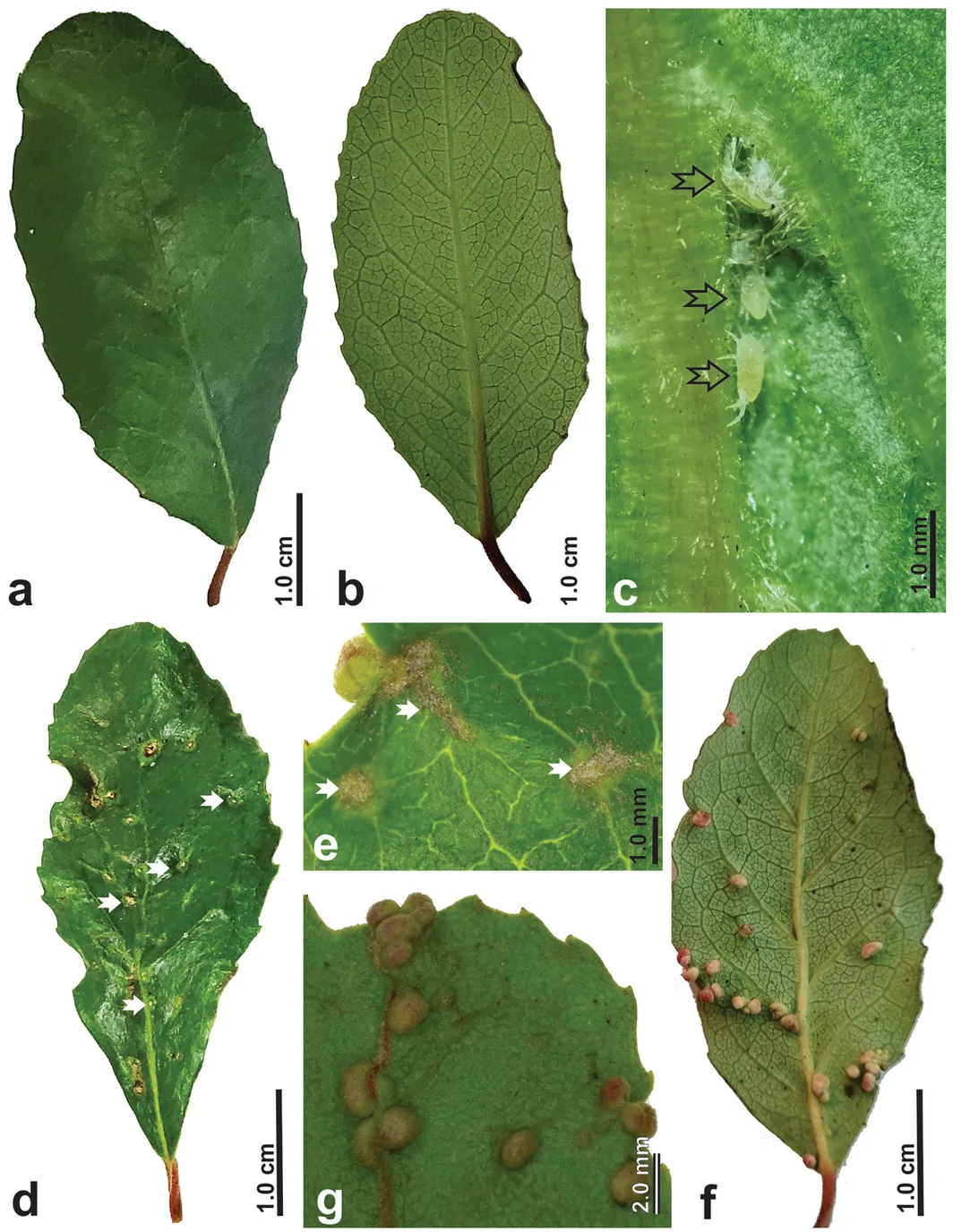
Leaves of *Crinodendron patagua* without (a, b) and with galls (c–f). (a) Adaxial (upper) surface of a non‐galled leaf. (b) Abaxial (underside) surface of a non‐galled leaf. (c) Adult Eriophyidae individuals (black arrows) on the abaxial surface during the gall induction process. (d) Adaxial surface of a leaf bearing numerous galls (white arrows). (e) Close‐up of the trichomes on the adaxial surface (white arrows). Note the leaf deformation caused by gall formation at the margin. (f) Abaxial surface of a leaf with multiple globoid galls, occurring singly or in clusters and always associated with veins. Note the reddish coloration of the galls. (g) Details of galls occurring along the leaf veins.

The leaves of *C. patagua* are obovate with an obtuse apex and serrated margins, characterized by shallow, regularly spaced teeth (Fig. 1a,b). Venation is pinnate, with a prominent midrib and well‐defined secondary veins (Fig. 1a,b). The adaxial surface is mostly glabrous and dark green (Fig. 1a), whereas the abaxial surface is hairy and light green (Fig. 1b). The petiole is short and reddish at the base.

Unidentified Eriophyidae induce galls on the abaxial side of *C. patagua* leaves (Fig. 1c), where colonies initially appear in small depressions on the leaf blade, covered with numerous white trichomes (Fig. 1d,e). These galls protrude towards the abaxial side as globoid, hairy structures that are light green with a reddish colour (Fig. 1f,g) and host numerous mites internally. Galls are distributed throughout the leaf blade but are consistently associated with veins (Fig. 1f,g). They may occur singly or in clusters (Fig. 1f,g). When present in large numbers, especially along the leaf margins, they can cause noticeable deformation of the leaf (Fig. 1e–g).

The leaves sampled in this study had an average of 24 galls per leaf, ranging from a minimum of 6 galls per leaf to a maximum of 66 galls per leaf. However, this did not affect the leaf area of *C. patagua*. In contrast, dry mass was, on average, 22.2% greater in galled leaves than in non‐galled leaves (Table 3). Additionally, leaf mass per area, an index of sclerophylly, was not affected by gall induction (Table 3).

**Table 3:** Comparison of leaf morphological traits between galled and non‐galled leaves of *C. patagua*.

Parameters	Types of leaves		χ^2^	df	*P* value
	Non‐galled leaves	Leaf galls			
Leaf area (m^2^)	0.09 ± 0.02	0.09 ± 0.02	0.45	1	> 0.05
Dry mass (g)	0.09 ± 0.02	0.11 ± 0.03	4.30	1	**< 0.01**
Leaf mass per area (g m^−2^)	1.14 ± 0.26	1.25 ± 0.31	6.96	1	> 0.05

Linear mixed models were used for leaf area and generalized linear mixed models with a gamma distribution and identity or log link were used for leaf mass per area and dry mass, respectively. In all the models, the plant individual was included as a random effect (n = 5). The data are presented as the means ± standard deviations. Significant differences (*P* ≤ 0.05) are indicated in bold.

### Anatomical description of leaves and galls

**Fig. 2:**
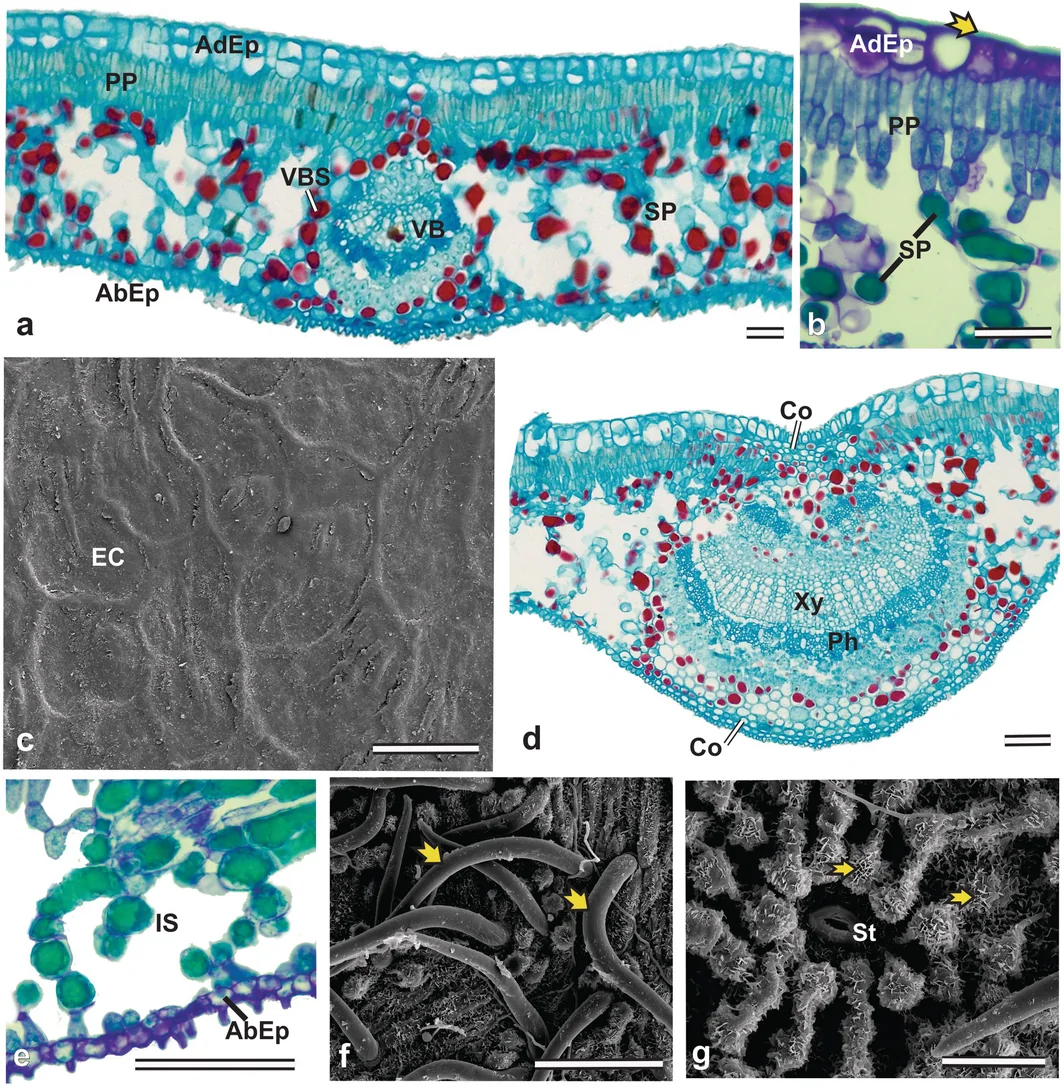
Anatomical features of *C. patagua* leaves. (a) Leaf blade with a dorsiventral mesophyll and a secondary vein. (b) Close‐up of the mesophyll showing the biseriate adaxial epidermis covered by a thick cuticle and two layers of palisade parenchyma. (c) Frontal view of the adaxial epidermis; note the absence of trichomes (glabrous surface). (d) The midrib is composed of two small vascular bundles and one large arch‐shaped vascular bundle surrounded by phloem fibres and layers of collenchyma projecting towards the abaxial surface. (e) Spongy parenchyma with abundant spaces bordered on the abaxial side by the papillose epidermis and numerous trichomes. (f) Frontal view of the abaxial epidermis showing filamentous unicellular trichomes. (g) Close‐up of the abaxial epidermis in frontal view, showing sunken stomata covered by ornate epicuticular waxes and surrounded by filiform trichomes. Images were obtained using light microscopy in transverse sections (a, b, d, e) and SEM (c, f, g). AbE, abaxial epidermis; AdE, adaxial epidermis; Co, collenchyma; Cu, cuticle; EC, epidermal cells; EpW, epicuticular waxes; IS, intercellular space; Ph, phloem; PP, palisade parenchyma; SP, spongy parenchyma; St, stomata; Tr, trichomes; VB, vascular bundle; VBS, vascular bundle sheath; Xy, xylem. Scale: 100 μm (a, c, g); 50 μm (b, d, e); 20 μm (f).

The adaxial epidermis of *C. patagua* leaves appears to be composed of one or two layers, as suggested by their topographical continuity along the leaf blade (Fig. 2a,b). The epidermal cells are large, rounded‐to‐polyhedral in cross‐section and characterized by large vacuoles (Fig. 2a,b). The epidermal cells exhibit dark violet metachromatic staining with toluidine blue at pH 4.7 (Fig. 2b). This reaction is consistent with a high density of anionic radicals, which are typically associated with pectin‐rich cell walls and mucilaginous protoplasts in plant tissues. Although the adaxial epidermis is mostly glabrous (Fig. 2a–c), some filiform trichomes are observed on the midrib. This epidermis is covered by a smooth, thick cuticle without ornamentation (Fig. 2b,c). The leaf is hypostomatic, and the mesophyll is dorsiventral, with 2–3 layers of tubular cells forming palisade parenchyma (Fig. 2a,b). The spongy parenchyma consists of up to seven layers of polyhedral cells, with very large and abundant intercellular spaces and numerous idioblasts filled with substances that stain red with safranin (Fig. 2a,d) and brownish green with toluidine blue at pH 4.7 (Fig. 2b), suggesting the presence of polyphenolic compounds.

The midrib is convex on the adaxial surface and covered by anticlinally elongated papillose cells in continuity with the large rounded‐to‐polyhedral pectic cells of the adaxial epidermis covering the leaf blade (Fig. 2d). Two to three layers of collenchyma occur under the adaxial epidermal surface, followed by 2–5 layers of homogeneous parenchyma cells, some with vacuoles stained red by safranin (Fig. 2d). The vascular system is formed by two smaller collateral vascular bundles at the adaxial portion and a prominent arch‐shaped collateral bundle abaxially, with the phloem faced by 2–5 layers of phloem fibres (Fig. 2d). The bundle sheath of the vascular bundles is parenchymatous (Fig. 2a,d). In the minor veins, the bundle sheath extends to both epidermal surfaces (Fig. 2a). The midrib is projected to the abaxial surface with a concave, rounded contour (Fig. 2d). The phloem fibres are followed by 2–4 layers of homogeneous parenchyma cells with vacuoles stained red by safranin, with some cells exhibiting thin secondary walls (Fig. 2d). Adjacent to these layers, there are 2–3 layers of collenchyma cells, all of which are covered by papillose epidermal cells. The abaxial epidermis consists of a single layer of papillose cells (Fig. 2e). The thickened walls are consistent with a high pectic composition, similar to that of the adaxial epidermis (Fig. 2e). Abaxial trichomes are abundant, unicellular and filamentous, covering the veins and interveinal spaces (Fig. 2f). Compared with ordinary epidermal cells, the cuticle on the abaxial epidermis is thick and ornamented and is particularly abundant around the stomata, which are slightly sunken (Fig. 2f,g).

**Fig. 3:**
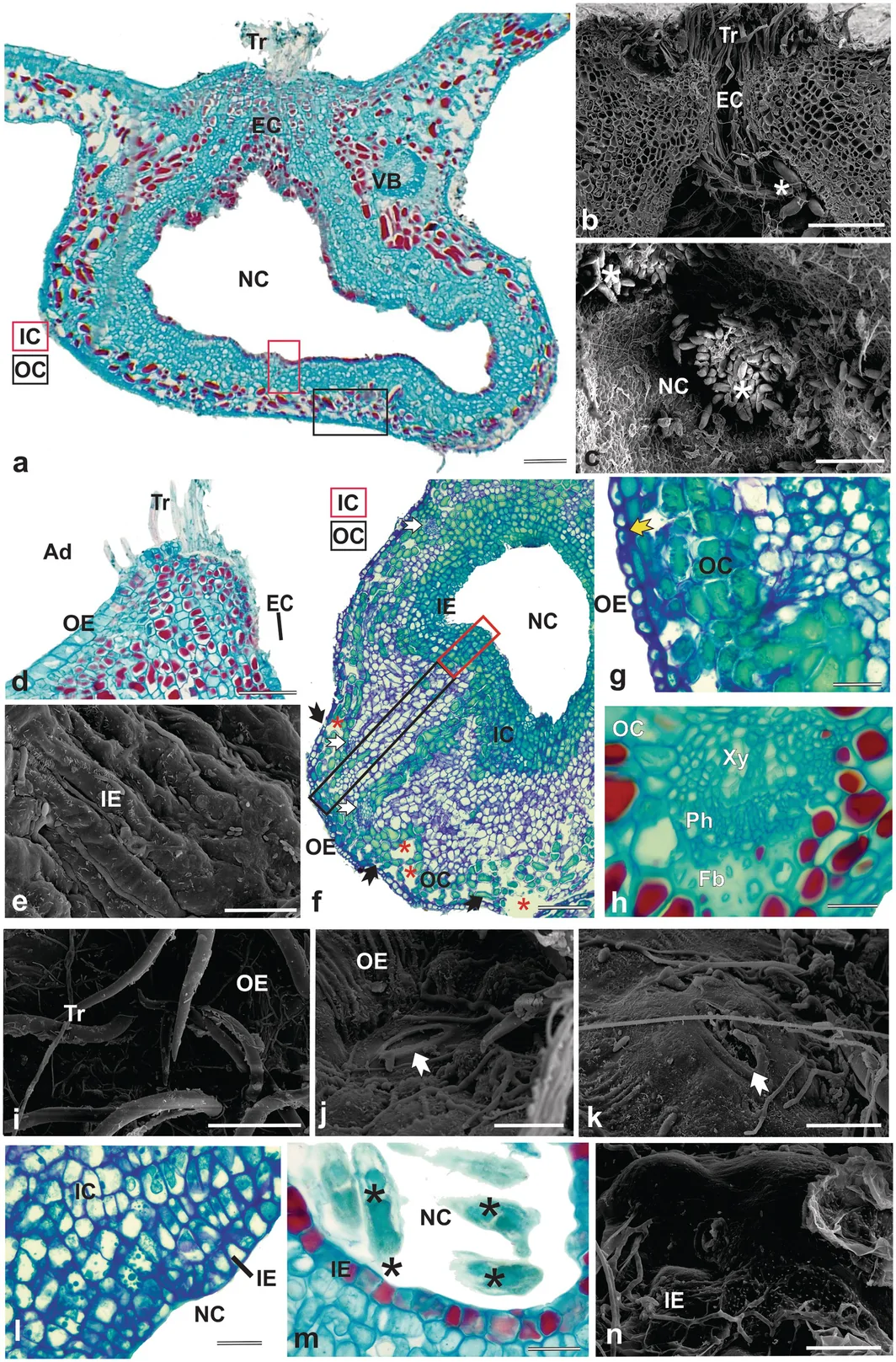
Anatomical features of galls induced by Eriophyidae on *C. patagua*. (a) General view of a semiclosed gall in cross section. Note the distinction between the inner cortex (red box, IC) and the outer cortex (black box, OC). (b) Detail of the gall opening, showing the invagination of the adaxial epidermis that forms the innermost layer of the gall. The white asterisk indicates the presence of mites. (c) General view of the inner epidermis covering the gall chamber, with numerous mites visible in this area (white asterisks). (d) Close‐up of the gall opening, showing numerous trichomes and the characteristics of the adaxial epidermis in cross section. (e) Frontal view of the outer epidermis covering the outer portion of the gall on the adaxial leaf side. (f) Transversal section of a gall stained with toluidine blue, illustrating the differentiation between the inner (red box) and outer cortex (black box). Note the intercellular spaces in the OC (red asterisks) associated with stomata (black arrows). The white arrows indicate vascular bundles. (g–k) Detail of the outer cortex of the gall: (g) Polyhedral cells stained with toluidine blue, with stomata associated with an intercellular space (yellow arrow). Note that the walls of the outer epidermal cells were stained blue with toluidine blue; (h) Vascular bundle located in the OC. (i–k) Filiform trichomes on the abaxial epidermis of the gall and stomata. The stomata appear sunken (j) or raised (k) relative to the ordinary epidermal cells. (l–n) Details of the inner cortex of the gall in cross section: (l) Details of the nymphal chamber, inner epidermis and parenchyma. Note that the walls of the epidermal cells stained blue with toluidine blue; (m) Detail of the nymphal chamber and inner epidermis with cell vacuoles intensely stained with safranin. The black asterisks indicate the presence of mites; (n) Frontal view of the inner epidermis surrounding the nymphal chamber. Images were obtained using light microscopy in transverse sections (a, d, f‐h, l, m) and scanning electron microscopy (b, c, e, i, j, k, n). EC, exit channel; Fb, fibres; IC, inner cortex; IE, inner epidermis; NC, nymphal chamber; OC, outer cortex; OE, outer epidermis; Ph, phloem; Tr, trichomes; VB, vascular bundle; Xy, xylem. Scale bars: 250 μm (c); 200 μm (a, b); 100 μm (d, f); 50 μm (g, h, j, k, l); 20 μm (e, i, m, n).

The galls induced by Eriophyidae on *C. patagua* leaves are composed of different cell types that form two distinctive gall regions, the outer and inner gall cortices (Fig. 3a). The adaxial epidermis of the leaf invaginates to form the inner epidermis lining the nymphal chamber (Fig. 3a–c), resulting in a semiclosed gall structure whose opening is partially obstructed by numerous newly formed unicellular trichomes (Fig. 3b,d). Numerous nymphs are housed within the gall (Fig. 3b,c). The epidermis covering the outer surface of the gall on the adaxial leaf side is uniseriate and composed of cells with a quadrangular outline in cross section, with thickened and slightly convex periclinal walls (Fig. 3d). In frontal view, the cells exhibit a slightly undulating pattern characterized by their polygonal shape and clearly defined anticlinal walls (Fig. 3e).

The outer gall epidermis on the abaxial leaf side is continuous with the abaxial epidermis of the non‐galled portion of the leaf blade (Fig. 3a) and is composed of rectangular or slightly papillose cells on the outer surface of the galls (Fig. 3f,g) and unicellular, filamentous trichomes (Fig. 3i). The outer epidermis was stained blue to purple with toluidine blue, suggesting the presence of pectocellulosic cell wall thickening (Fig. 3g). The stomata, which appear hypertrophied, are scarce and show no uniform distribution pattern (Fig. 3j–k). Some stomata are slightly sunken (Fig. 3j), whereas others protrude above the level of the surrounding epidermal cells (Fig. 3k).

Under the outer gall epidermis, the outer gall cortex (Fig. 3a,f) is composed of 4–7 layers of homogeneous parenchyma with polyhedral cells, which are visually larger than the inner gall parenchyma cells and have vacuoles filled with substances stained with safranin (Fig. 3a) and toluidine blue (Fig. 3f,g). These cells resemble the spongy parenchyma of the leaf but are more compact, although intercellular spaces, which are associated mainly with stomata (Fig. 3f,g), are still present. Some vascular bundles are present in the outer cortex (Fig. 3h) and connect to the nymphal chamber via elongated parenchyma cells (Fig. 3a,f). Other vascular bundles extend from the leaf into the gall structure (Fig. 3a).

The inner gall cortex is formed by inner layers of parenchyma cells, which are visually smaller than the outer parenchyma cells, followed by the inner epidermis in contact with the gall chamber (Fig. 3a,f,l). The inner gall parenchyma is continuous with the palisade parenchyma of the non‐galled leaf portions (Fig. 3a) and is composed of 2 to 7 layers of tightly packed cells with minimal intercellular spaces (Fig. 3f,l,m). Some of these cells possessed abundant cytoplasmic contents (Fig. 3f,l,m). The innermost cellular layer surrounding the nymphal chamber corresponds to the invaginated adaxial epidermis, which constitutes the feeding site for the nymphs (Fig. 3a,c,f). In cross section, these inner epidermis cells exhibit a rectangular shape with dense cytoplasm and occasionally contain inclusions that stain brownish green with toluidine blue at pH 4.7 (Fig. 3l) and red with safranin (Fig. 3m), suggesting the presence of phenolic compounds. Their cell walls appear thickened and stain blue with toluidine blue (Fig. 3l), suggesting differences in cell wall composition between galled and non‐galled tissues. In frontal view, these epidermal cells display a markedly undulating surface with irregular anticlinal contours (Fig. 3n).

### Cytohistometric results

Statistical comparisons of the cytohistometric variables revealed that only the thickness of the adaxial epidermis, the area of the epidermal cells (both adaxial and abaxial) and the thickness of the abaxial epidermal cell walls were affected by the gall‐inducing activity of the eriophyid mites on *C. patagua* leaves (Table 4). However, the direction of the changes was not consistent across the variables. On the adaxial side, both affected variables decreased in galled tissue in the cells surrounding the gall chamber, with the adaxial epidermal thickness reduced by 45.8% and the area of adaxial epidermal cells decreased by 59.8%. Conversely, on the abaxial side, where the cells compose the outer gall covering, the area of epidermal cells increased by 1.7‐fold and the thickness of their cell walls increased by 1.5‐fold. No differences were detected in the thickness of the adaxial cell walls, mesophyll or abaxial epidermis between galled and non‐galled leaves (Table 4).

**Table 4:** Comparison of cytohistometric traits between galled and non‐galled leaves of *C. patagua*.

parameters of non‐galled leaves/galls	types of leaves		χ^2^	df	*P* value
	non‐galled leaves	leaf galls			
Adaxial/inner epidermis thickness (μm)	56.30 ± 11.30	30.50 ± 10.5	60.41	1	**< 0.001**
Adaxial/inner epidermal cell area (μm^2^)	2025.00 ± 614.00	813.00 ± 381.00	2.75	1	**< 0.001**
Adaxial/inner epidermal cell wall thickness (μm)	3.45 ± 0.91	2.81 ± 1.35	0.78	1	> 0.05
Leaf mesophyll/gall cortex thickness (μm)	327.00 ± 43.30	313.00 ± 49.9	0.37	1	> 0.05
Abaxial/outer epidermis thickness (μm)	19.30 ± 2.520	20.30 ± 5.94	15.87	1	> 0.05
Abaxial/outer epidermal cell area (μm^2^)	388.00 ± 88.50	666.00 ± 250.00	47.25	1	**< 0.001**
Abaxial/outer epidermal cell wall thickness (μm)	1.83 ± 0.33	2.77 ± 1.27	25.91	1	**< 0.001**

Linear mixed models were used to analyse adaxial epidermis, mesophyll and abaxial epidermis thickness and the adaxial epidermal cell area. Generalized linear mixed models with a gamma distribution and a log link function were used for adaxial epidermal cell wall and abaxial epidermal cell wall thickness and abaxial epidermal cell area. In all the models, individual plants were included as a random effect (n = 5). The data are presented as the means ± standard deviations. Significant differences (*P* ≤ 0.05) are indicated in bold.

### Histolocalization of primary and secondary metabolites

**Fig. 4:**
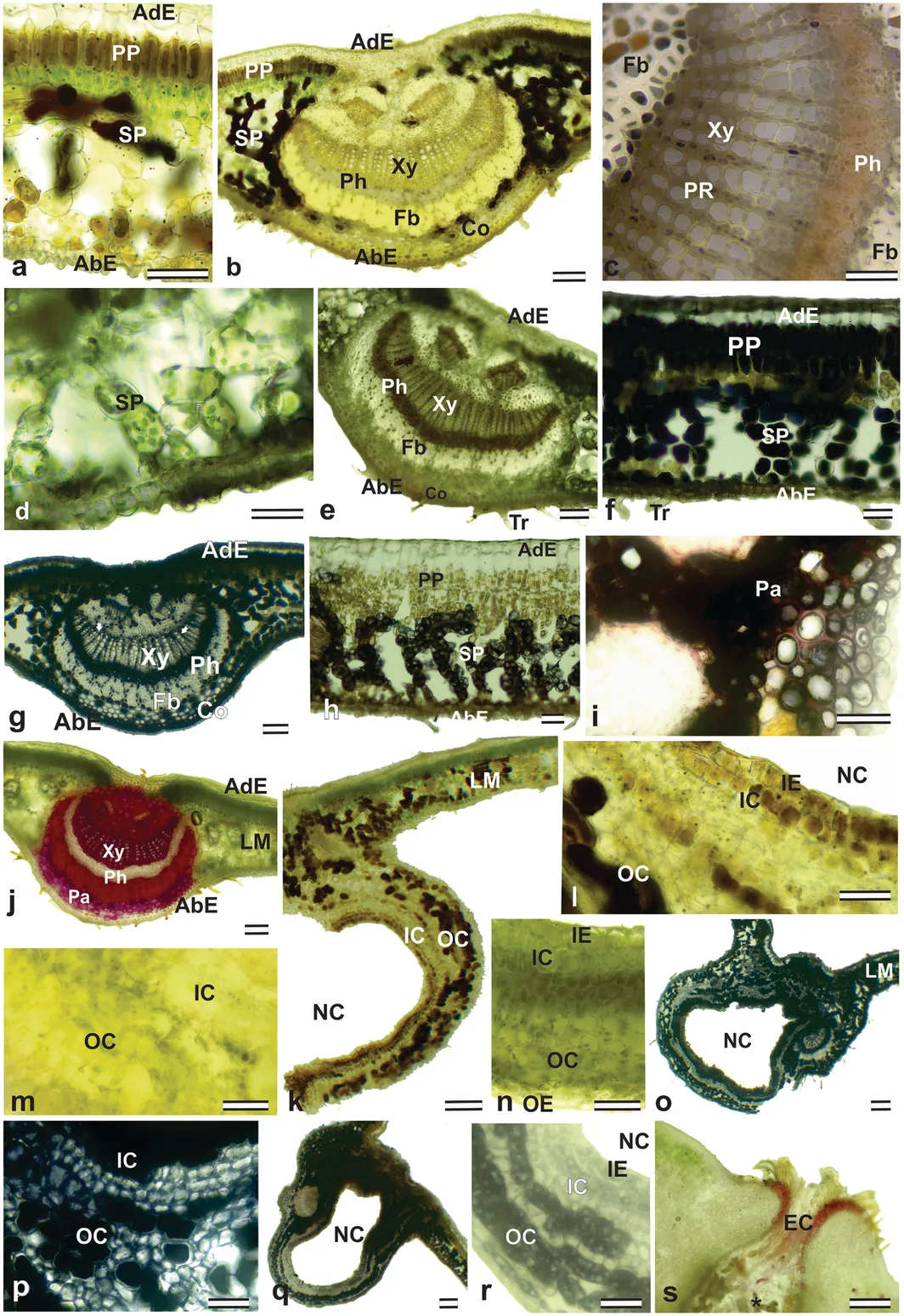
Histolocalization of primary and secondary metabolites in non‐galled leaves (a–j) of *C. patagua* and in leaf galls (k–s) induced by Eriophyidae. Detection of reducing sugars (a, b, k, l), starch (c, m), proteins (d, e, n), phenols (f, g, o, p), flavonols (h, i, q, r) and lignin (j, s). In g, the white arrows indicate the parenchyma rays, and in s, the asterisk indicates the presence of mites. AbE, abaxial epidermis; AdE, adaxial epidermis; Co, collenchyma; EC, exit channel; Fb, fibre; IC, inner cortex; LM, leaf mesophyll; NC, nymphal chamber; OC, outer cortex; Pa, parenchyma; Ph, phloem; PP, palisade parenchyma; PR, parenchyma ray; SP, spongy parenchyma; Tr, trichomes; VB, vascular bundle; Xy, xylem. Scale bars: 200 μm (g, k, o, q); 100 μm (b, e, f, h, j); 50 μm (a, c, d, i, l, m, n, p, r, s).

The non‐galled leaves of *C. patagua* showed positive histochemical reactions for reducing sugars, starch, proteins, phenols and soluble flavonols but in different tissues (Fig. 4a–j). Reducing sugars were detected in palisade and spongy parenchyma cells, as well as in some collenchyma cells located on the abaxial side of the midrib (Fig. 4a,b). Starch grains were detected only in some cells of the vascular fibres and xylem rays of the midrib (Fig. 4c). Proteins were observed as precipitates in the chloroplasts of both palisade and spongy parenchyma cells (Fig. 4d), as well as in the collenchyma, phloem fibres and xylem rays of the midrib (Fig. 4e). Phenols accumulated in the cell walls of the epidermis on both surfaces; in the vacuoles of palisade and some spongy parenchyma cells; and in the phloem, phloem fibres, xylem rays and collenchyma cells of the midrib (Fig. 4f,g). Flavonols were detected in the vacuoles and cell walls of spongy parenchyma (Fig. 4h) and collenchyma cells (Fig. 4i). Lignin was detected in the cell walls of xylem elements, phloem fibres and some cortical parenchyma cells of the midrib (Fig. 4j), as well as in the xylem and phloem fibres of the vascular bundles in minor veins (not shown).

The galls of Eriophyidae induced on *C. patagua* leaves tested positive for reducing sugars, starch, proteins, phenols and flavonols, with a differential distribution among their tissues (Fig. 4k–s). Reducing sugars were detected in the epidermis lining the nymphal chamber and in some cells of the inner and outer cell cortex (Fig. 4k,l). Starch grains were scarce and were observed only in some cells of the outer cortex (Fig. 4m). Proteins were detected as diffuse signals in both the inner and outer cortex, as well as in the cell layers surrounding the nymphal chamber (Fig. 4n). Phenols were detected in the cell walls of both the inner and outer cortex and in the vacuoles of some of these cells (Fig. 4o,p). Flavonols were detected in the cell walls of the inner and outer cortex and in the vacuoles of certain cells in these tissues (Fig. 4q,r). The vascular bundles within the galls exhibited a lignification pattern similar to that of the non‐galled leaves, with lignin deposited in the xylem elements and phloem fibres (not shown). A positive reaction for lignin was also observed in the walls of the exit channel cells and in the overlapping trichomes (Fig. 4s).

### Primary and secondary metabolite contents

**Fig. 5:**
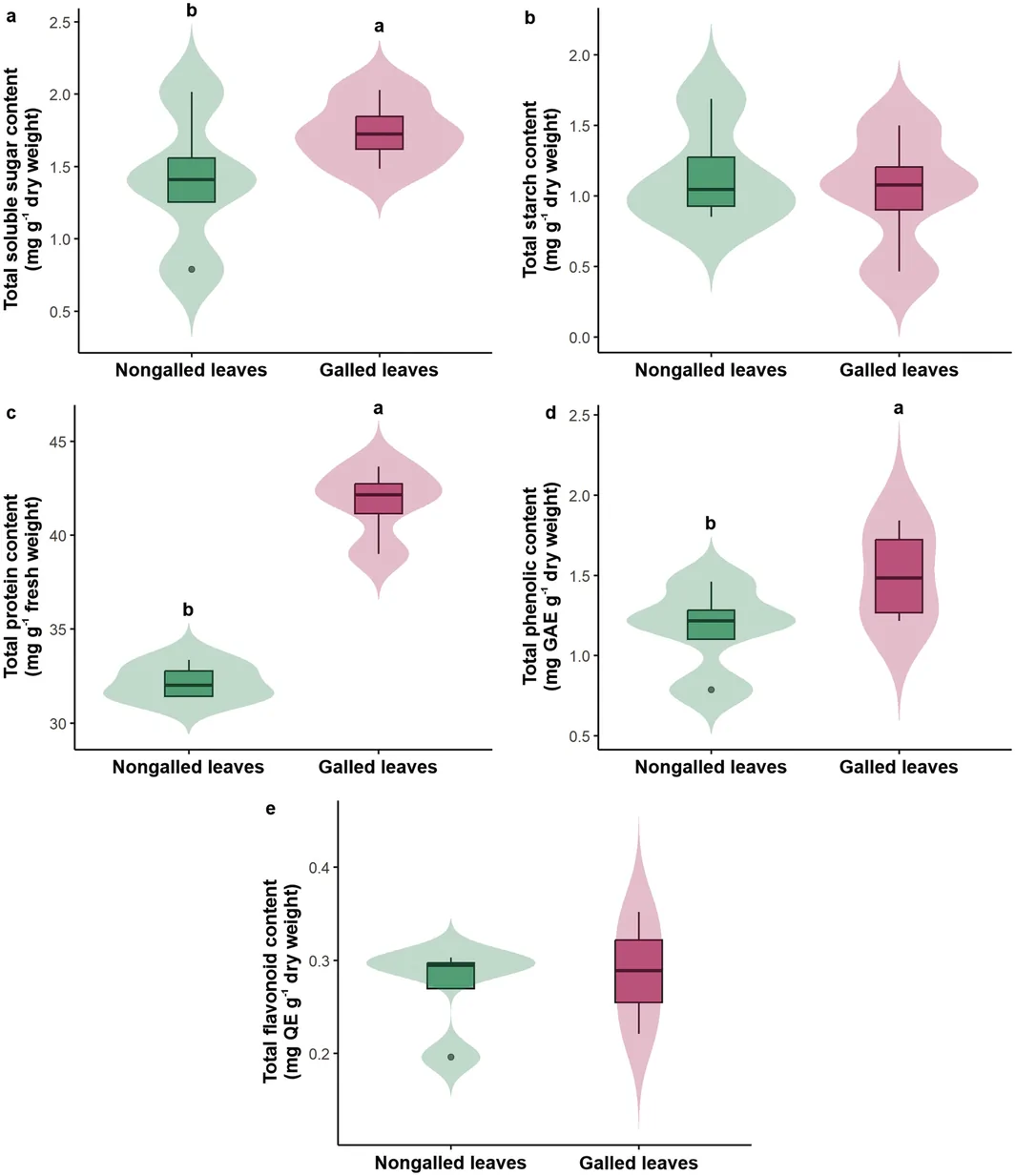
Primary and secondary metabolite contents in non‐galled leaves (green) and galls (pink) induced by Eriophyidae on *C. patagua* leaves. (a) Total soluble sugars. (b) Total starch. (c) Total proteins. (d) Total phenolics. (e) Total flavonols. The distinct letters on the boxplots in the same graph indicate significant differences when linear mixed models (LMMs) or generalized linear mixed models (GLMMs) are used, with a significance level of 5% (*P* ≤ 0.05).

The establishment of Eriophyidae on the leaves of *C. patagua* increased the contents of reducing sugars (χ^2^ = 19.81, df = 1, *P* < 0.001) (Fig. 5a), whereas the starch content was not affected (χ^2^ = 0.24, df = 1, *P* > 0.05) (Fig. 5b). Protein levels significantly increased (χ^2^ = 518.46, df = 1, *P* < 0.001) (Fig. 5c), as did total phenols (χ^2^ = 19.51, df = 1, *P* < 0.001) (Fig. 5d). In contrast, the flavonoid content remained unchanged (χ^2^ = 1.89, df = 1, *P* > 0.05) (Fig. 5e).

## DISCUSSION

The development of mite‐induced galls on *C. patagua* leaves involves selective reprogramming of the host's structural and biochemical traits: while some sclerophyllous characteristics are maintained or even reinforced, others remain unchanged or are not expressed. This differential modulation of sclerophyllous traits indicates that the initial hypothesis is only partially supported. Histochemical and quantitative analyses further demonstrated that galls function as nutrient‐rich microenvironments, reflecting a targeted reprogramming of host tissues that favours both the nutrition and protection of gall inducers and their progeny.

### Protective functions of gall traits in a sclerophyllous context

The evergreen, thick leaves of *C. patagua* exhibit typical sclerophyllous traits, including a thick cuticle, abundant abaxial pubescence, sunken stomata, a stratified epidermis, a compact palisade parenchyma and pronounced sclerification, particularly around the vascular bundles (Read *et al*. 2016; Guerra & Scremin‐Dias 2018). These anatomical features are associated mainly with water conservation during prolonged dry summers. Additionally, pectin‐thickened cell walls in the adaxial and abaxial epidermis, along with the accumulation of mucilaginous substances, function as hygroscopic adaptations that help maintain hydration under dry conditions (Shin *et al*. 2021). Pectins have been implicated in drought stress tolerance in a wide range of plant systems, mainly through their ability to form hydrated gels in the cell wall and modulate water relationships (Konno *et al*. 2003; Leucci *et al*. 2008; Chen *et al*. 2015; Tenhaken 2015). In the galls of *C. patagua*, these traits were reinforced, specifically by the greater presence of lignified trichomes and the localized accumulation of polyphenols, which may further enhance the shielding of the inducer against the high irradiance and desiccation typical of Mediterranean‐type environments. However, whether these biochemical and structural adjustments directly translate to increased gall‐inducer survival remains to be experimentally investigated.

Some sclerophyllous traits of *C. patagua* leaves are retained or even enhanced within galls. For example, the maintenance of leaf mass per area in galled leaves may reflect a balance between mite‐induced alterations involving both hypotrophy and hypertrophy, particularly in epidermal cells. While the adaxial epidermis has a reduced cell size and thickness (hypotrophy), the abaxial epidermis has thickened cell walls and increased cell area (hypertrophy). Additionally, mite‐induced changes in tissue composition, such as parenchyma compaction and homogenization, along with a reduction in intercellular spaces and localized lignification, result in greater dry mass. However, these modifications did not alter the LMA because of the preservation of the leaf area.

Trichomes were abundant in the abaxial epidermis of both galled and non‐galled leaves. However, in the adaxial epidermis, trichomes are typically confined to the midrib region in non‐galled leaves, whereas mite‐induced galls exhibit a profusion of unicellular (some lignified) trichomes around the gall ostiole. Trichomes may provide physical protection against natural enemies, help maintain humidity within the gall, buffer solar radiation, including UV radiation (Moura *et al*. 2008, 2009; Michalska *et al*. 2010; Amada *et al*. 2019; Rezende *et al*. 2020; Costa *et al*. 2022; Costa 2025), and thereby protect gall‐inducing organisms in Mediterranean environments (Guedes *et al*. 2018a, 2018b, 2022; Guedes, Henríquez, *et al*. 2024). Additionally, in *C. patagua*, the thickened walls of hypertrophied abaxial epidermal cells covering the gall cortex may increase resistance to abiotic stress (Xiong *et al*. 2015) and function as an apoplastic water reservoir (Nedukha 2022), representing an adaptive response to the seasonally dry Mediterranean climate.

Lignin deposition was another notable feature observed specifically in the epidermal cells of the gall cortex and trichomes near the ostiole. This reinforcement likely enhances the structural integrity of gall opening and ensures a stable environment for mite development, as reported in other mite‐induced galls (Kane *et al*. 1997; Chetverikov *et al*. 2015; Guedes *et al.* 2022; Guedes, Henríquez, *et al*. 2024).

High concentrations of secondary metabolites, especially polyphenols, have been described as sclerophyllous traits related to defence mechanisms (Read *et al*. 2016; Guerra & Scremin‐Dias 2018). Phenolic compounds, in particular, may accumulate in the cell walls to reinforce structural support or be stored in vacuoles, functioning in both cases to counter oxidative stress induced by gall development and abiotic stressors, such as high irradiance and low water availability in Mediterranean environments (Guedes *et al*. 2022; Parvin *et al*. 2022), where *C. patagua* galls develop. An increase in polyphenolic content has been reported in eriophyid‐induced galls, such as those in *Miconia ibaguensis–*Eriophyidae (Ferreira *et al*. 2018) and in *Tilia platyphyllos–Eriophyes tiliae* systems (Guedes *et al*. 2023), both of which are associated with protection against oxidative stress and UV radiation. Our results align with these findings, as both the accumulation sites—in cell walls and vacuoles—and the increase in total polyphenolic content were evident in *C. patagua* galls. Although flavonoid levels did not increase, their specific localization in the outer gall layers suggests a protective role, as previously reported in other gall systems (Eshwarappa *et al*. 2015; Kuster *et al*. 2019; Guedes *et al.* 2022, 2025; Guedes, Aguilera, *et al*. 2024).

### *Crinodendron patagua* galls as nutrient‐rich structures

Some anatomical traits of galls induced by eriophyid mites suggest that not all scleromorphic characteristics are preserved within the galls. Differences in toluidine blue staining patterns at pH 4.7 indicate variations in cell wall properties between galled and non‐galled tissues. These differences likely reflect changes in cell wall composition; however, given the non‐specific nature of toluidine blue staining, further specific histochemical or immunohistochemical analyses are needed to confirm the presence and distribution of particular polysaccharides. Beyond these preliminary observations, alterations in cell wall composition have been widely reported in both insect‐ and mite‐induced galls and are associated with functional changes in redifferentiated cells (Ferreira *et al*. 2020), contributing to both protective and nutritional roles (Bragança *et al*. 2020; Costa *et al*. 2022; Souza *et al*. 2024; Guedes *et al*. 2025).

In contrast to the thickened outer walls of the hypertrophied abaxial epidermal cells, the reduced thickness of the adaxial epidermal cell walls—where eriophyid mites feed—likely represents a nutritional adaptation that facilitates stylet penetration. This thinning may increase access to nutrient‐rich compounds such as proteins and reducing sugars, reinforcing the nutritional strategy employed by eriophyid mites within galls. Mites penetrate host cells using their stylets, mechanically perforating cell walls composed primarily of cellulose and hemicellulose (Petanović & Kielkiewicz 2010; Klimov *et al*. 2018). During this process, they secrete saliva containing cell wall‐modifying enzymes that degrade components of the middle lamella and primary wall (Westphal & Manson 1996; Lillo *et al*. 2018; Yang *et al*. 2023). This enzymatic activity enables stylet insertion and sustained feeding, as demonstrated for *Aceria pallida* Keifer, 1969 (Acari: Eriophyidae) (Yang *et al*. 2023). As a result, both cytoplasmic contents—rich in proteins and reducing sugars—and wall‐derived compounds, mainly hemicelluloses, become accessible to mites. These findings support the idea that the modified cell walls in *C. patagua* galls are not only structurally reorganized but also biochemically reprogrammed to facilitate nutrient acquisition. Notably, xyloglucans, a major class of hemicelluloses, are considered the principal storage polysaccharides in plant cell walls (Reid 1985; Hoch 2007; Marcus *et al*. 2010). Moreover, epidermal cell walls contribute significantly to the structural and functional properties of galls (Costa 2025), although their role in eriophyid‐induced galls remains underexplored (Ferreira *et al*. 2025).

In a sclerophyllous context, where leaves are typically carbon‐dense and nutritionally restrictive, such local modifications represent a marked override of host nutritional constraints. Accordingly, the accumulation of reducing sugars and proteins in the nutritive cells lining the larval chamber, along with the increased concentrations of these metabolites in galled leaves, confirms that eriophyid‐induced galls on *C. patagua* function as nutrient‐rich structures. While this characteristic may be partly influenced by the Mediterranean‐type environment of central Chile, similar patterns have been observed in eriophyid galls from diverse ecological regions. For example, mite galls on *M. ibaguensis* and *M. notabilis* (Ferreira *et al*. 2017; Freitas *et al*. 2023), *Avicennia schaueriana* Stapf & Leechm. ex Moldenke (Nobrega *et al*. 2023), *Vitis vinifera* L. (Guedes, Henríquez, *et al*. 2024) and *T. platyphyllos* (Guedes *et al*. 2023) also accumulate proteins, lipids and reducing sugars in nutritive cells lining the larval chamber—the primary feeding site of gall‐inducing mites (Ferreira *et al*. 2025).

In contrast, starch was detected only in small amounts and was restricted to the outer cortex of the galls. This likely reflects a host‐specific trait, as the starch accumulation in the non‐galled leaves of *C. patagua* was low. The spatial distribution of starch within gall tissues varies and is influenced by factors such as the identity of the host plant, the gall‐inducing species and the morphology of the insect's feeding apparatus (Giertych 2025). Interestingly, the sites of starch accumulation differed between non‐galled leaves and galled leaves. In non‐galled leaves, starch was associated mainly with vascular tissues, possibly related to its mobilization towards sink organs. However, in galls, starch was localized in the outer cortex. In both mite‐induced galls and insect‐induced galls, the presence of starch and other metabolites in peripheral gall layers is associated with storage tissues (Ferreira *et al*. 2019) that contribute to the structural maintenance of the gall (Dardeau *et al*. 2014; Giertych 2025).

Furthermore, the presence of starch in the outer cortex suggested that the galls may retain some photosynthetic activity. This possibility is supported by the observation of stomata connected to substomatal chambers and intercellular spaces in close proximity to starch‐accumulating cells, potentially helping to prevent hypercarbia and hypoxia within the gall microenvironment (Costa 2025). Nevertheless, the hypothesis concerning the photosynthetic capacity of *C. patagua* galls remains to be confirmed, since our study revealed the presence of stomata with altered disposition and apparently smaller proportions than in non‐galled leaves. Taken together, these results support the nutritional component of our initial hypothesis, demonstrating that eriophyid mites are able to locally reprogram typically low‐nutrient sclerophyllous leaves into nutrient‐enriched gall tissues. However, whether such nutritional enrichment translates to enhanced mite performance or survival remains to be experimentally tested.

## CONCLUSION

Together, our results partially support the hypothesis that eriophyid mites induce targeted anatomical changes that modify or reinforce specific sclerophyllous traits. In parallel, these changes promote the accumulation of nutrient‐rich tissues, particularly proteins and reducing sugars, ensuring both protection and nutritional support for mite development under the constraints of a Mediterranean‐type environment. These findings not only deepen our understanding of plant–mite interactions in sclerophyllous systems but also highlight how gall inducers can exploit host traits to cope with environmental stress. Future research should explore whether such strategies are conserved across eriophyid species and Mediterranean‐type ecosystems.

## AUTHOR CONTRIBUTIONS

LMG and BGF contributed to the experimental design, data collection, data analysis and manuscript composition. LRC, EG, FRV and JFPO performed the experimental collection and evaluation, spectrometric analyses and data collection. NA was the supervising author and was involved in concept formation, experimental design and manuscript editing. JFL oversaw the data analysis, statistical processing and manuscript editing.

## Supporting information


**Fig. S1.** Study site. (a) Map showing the province of Concepción (blue) within the Biobío Region, Chile. The yellow point indicates the approximate location of the Universidad de Concepción (UdeC, acronym in Spanish). (b) An adult *Crinodendron patagua* tree sampled at UdeC. (c) A lateral branch bearing both galled and non‐galled leaves.

**Data S2.** Methodological procedures.

## Data Availability

All the data generated or analysed during this study are included in this published article.
