# Supplementary material for: Sclerophyllous structural and chemical traits of mite‐induced galls on Crinodendron patagua (Elaeocarpaceae) leaves
Source: Plant Biol (Stuttg). 2026 Jun 19;28(6):2039–53. doi: 10.1111/plb.70246 (PMC13586649; doi:10.1111/plb.70246)
Supplement: Supplementary file 1 [file PLB-28-2039-s002.docx]

**SUPPORTING INFORMATION 2**

**Sclerophyllous structural and chemical traits of mite-induced galls on Crinodendron patagua (Elaeocarpaceae) leaves**

Lubia M. Guedes^1^, Lorena Rodríguez-Cerda^1^, Narciso Aguilera^1^, Elizabeth Grandón^1^, Filipe R. Valeriano^2^, Jéssica Ferreira de Lima^3^, Julia Fernandes Pimenta de Oliveira^2^, Bruno Garcia Ferreira^2^

^1^ Universidad de Concepción, Facultad de Ciencias Forestales, Laboratorio de Semioquímica Aplicada, Concepción, Chile

^2^ Universidade Federal do Rio de Janeiro, Instituto de Biologia, Departamento de Botânica, Rio de Janeiro, Brazil

^3^ Universidade Federal de Uberlândia, Instituto de Biologia, Programa de Pós-graduação em Ecologia, Conservação e Biodiversidade, Uberlândia, Brazil

**Methodological procedures**

**Anatomical characterization of nongalled and galled leaves**

The anatomical description of galled and nongalled leaves of *C. patagua* was conducted on samples collected from the sixth and seventh nodes of five different trees (n = 5 per organ). Leaves were sectioned at their median third and fixed in either formalin-glacial acetic acid-70% ethanol (FAA; 1:1:18; v:v:v; Johansen 1940) for light microscopy or 2.5% glutaraldehyde (Coetzee & van der Merwe 1984) for electron microscopy studies. The samples fixed in FAA for 48 h were preserved in 70% ethanol, dehydrated in an ascending series of butyl alcohol, and immersed in a mineral oil-butanol mixture (1:1; v:v) before being embedded in Paraplast^®^ at 60°C in an oven. Paraplast blocks were transversely sectioned at 12 µm using a rotary microtome (Leica RM2125 RTS, Germany), and the sections were affixed to slides with Bissing's adhesive (Bissing 1974). The deparaffinized and hydrated sections were stained with 0.5% astra blue and 0.5% safranin (9:1; v:v) (Kraus & Arduin 1997) and mounted in Pertex^®^ mounting medium.

Another set of fragments of nongalled and galled leaves (n = 5 for each category), previously fixed in 2.5% glutaraldehyde, was also subjected to a gradual ethyl series for dehydration, embedded in Leica^®^ historesin and sectioned in a rotary microtome (5–8 μm). Transverse semithin sections were stained with 0.05% toluidine blue (O’Brien & McCully 1987) in acetate buffer, pH 4.7, and mounted via Pertex^®^. Toluidine blue O (TB) is a polychromatic cationic dye that binds to anionic radicals in plant tissues, displaying metachromasy according to the density and nature of these sites. Under acidic conditions (pH 4.0–4.7), purple-to-pink coloration (metachromasy) is indicative of strong binding to carboxylated polysaccharides, such as pectic acid in primary cell walls (O’Brien *et al.* 1964; Yeung 1998; Ribeiro & Leitão 2020). The prevailing blue coloration (orthochromasy) is consistent with lower pectin abundance in the wall. In contrast, prevailing green, greenish-blue, or bright blue coloration is associated with the presence of polyphenolic substances, such as lignin and tannins. Therefore, color differences in galled and nongalled tissues were interpreted as qualitative histochemical inferences following O’Brien et al. (1964), Yeung (1998), and Ribeiro & Leitão (2020).

Images for the anatomical description and histolocalization of primary and secondary metabolites (section 2.5) in nongalled and galled leaves were captured with a Leica microscope (Leica DM 500, Germany) equipped with a Leica digital camera (Leica ICC50 HD, Germany).

Samples of nongalled and galled leaves (n = 5 per condition) previously fixed in 2.5% glutaraldehyde were dehydrated in an increasing ethanol series and then critical point dried via a BALZERS UNION FL-9496 critical point dryer (Oerlikon Balzers Coating, Balzers, Liechtenstein). The fragments were subsequently glued to stubs with double carbon tape and coated with 25 nm of gold using a Leica EM CPD 300 sputtering device (Leica Microsystems, Wetzlar, Germany). The procedures were performed at the Laboratory of Cell Ultrastructure Hertha Meyer, Instituto de Biofísica Carlos Chagas Filho, Universidade Federal do Rio de Janeiro (UFRJ). Scanning electron microscopy (SEM) analyses were conducted with a Thermo Fisher Quattro S microscope (Thermo Fisher Scientific Inc., Waltham, USA) at the Centro Nacional de Biologia Estrutural e Bioimagem (CENABIO)/UFRJ.

**Histolocalization of primary and secondary metabolites**

Reducing sugars were detected by Fehling’s reagent, which produces a brown color upon reaction (Sass 1951). Starch was identified by the appearance of dark blue to black granules following treatment with Lugol’s reagent (Johansen 1940). Proteins were visualized as blue precipitates after staining with bromophenol blue (Baker 1958).

To detect soluble phenols, fragments approximately 1.5 cm wide from both nongalled and galled leaves (n = 5 per organ) were fixed for 48 h in a ferrous sulfate–formalin solution (4% formaldehyde, 10% FeSO₄·7H₂O in distilled water) and then freehand sectioned (Johansen 1940). The phenols were visualized as black precipitates. Similarly, additional 1.5 cm leaf segments were fixed for 72 h in caffeine-benzoate sodium solution (0.5% caffeine and 0.5% sodium benzoate in a 90:10 mixture of butyl alcohol and distilled water) and then sectioned for the detection of flavanols with *p*-dimethylaminocinnamaldehyde (DMACA) solution (1% p/v). Flavanols react with DMACA to form black or dark blue precipitates (Feucht *et al.* 1986). Lignin deposition in fresh sections was detected with acidified phloroglucinol (Wiesner reagent), which was prepared by mixing equal parts of 1% phloroglucinol in 95% ethanol and 18% HCl. The development of a pink colour was interpreted as a positive reaction (Johansen 1940).

**Quantification of soluble sugars and starch in leaf tissue**

***Total soluble sugar extraction*** (Rose *et al.* 1991): Soluble sugars were extracted with 2 mL of a methanol:chloroform:water mixture (12:5:3; v/v/v), sonicated and agitated for 5 min each. The samples were then centrifuged at 2200 rpm for 10 min, and the supernatant was carefully aspirated. The extraction was repeated, and the supernatants were pooled. Two milliliters of chloroform and 1.5 mL of deionized water were subsequently added to each sample, followed by centrifugation at 2200 rpm for 15 min (Dickson 1979).

***Starch solubilization*:** The pellet obtained from TSS extraction was oven-dried to remove residual chloroform and water. Once dried, it was resuspended in 0.5 mL of 47.5% ethanol and 3 mL of deionized water and then incubated in a 100°C water bath for 30 min (Rose *et al.* 1991). After cooling, 1 mL of amyloglucosidase solution prepared in 0.1 M sodium acetate buffer (pH 4.8) was added (160 µL of enzyme in 50 mL of buffer). The tubes were incubated overnight in a 45°C water bath.

***Quantification*:** For quantification, 200 µL of the TSS and glucose extracts were mixed with 400 µL of 2% phenol and 1 mL of concentrated sulfuric acid (Chow & Landhäusser 2004). The mixture was left in the dark for 10 min. Absorbance was measured in triplicate at 490 nm before the reaction was allowed to cool for 5 min. Blanks containing the enzyme for starch quantification and deionized water were included. The total soluble sugar and starch contents were calculated with standard curves of sucrose (1.5 mg mL^-1^) and glucose (1.0 mg mL^-1^), respectively, and expressed as milligrams per gram of dry weight (mg g⁻¹ DW).

**Quantification of soluble proteins**

Fresh leaf samples were macerated in liquid nitrogen, and 100 mg of plant material was mixed with 1 mL of extraction buffer (100 mM Tris-HCl, pH 7.6). The mixture was shaken for 10 min on ice. The samples were then immediately centrifuged at 10,000 rpm for 5 min at 4°C. Subsequently, 150 µL of the supernatant was mixed with 600 µL of Bradford reagent and incubated for 5 minutes to allow color development. The absorbance was measured at 595 nm against a blank without sample.

**Phenolic extract preparation**

Samples of nongalled and galled leaves (n = 4 trees), which were previously oven-dried at 40°C, were ground using a homogenizer for 2 min at 1110 rpm. The powdered samples were extracted with 80% aqueous methanol (0.1 g mL⁻¹), homogenized by vortexing (Vortex Genius 3, IKA^®^, Germany) for 1 min, subjected to ultrasonication (Power Sonic 603 Hst, South Korea) for 20 min, and then left in the dark at room temperature for 24 h. This extraction process was repeated three times. The filtrates from each extraction were pooled, filtered, and stored at 4°C.

***Quantification of total polyphenols:*** Total polyphenols were quantified with the Folin‒Ciocalteu method (Singh *et al.* 2016). For each reaction, 10 μL of extract was mixed with 20 μL of Folin reagent and 200 μL of distilled water. After standing for 5 min, 100 μL of 15% sodium carbonate was added, and the samples were incubated in the dark for 30 min. Absorbance was measured at 750 nm using a methanol blank. The total polyphenol concentration was calculated based on a gallic acid standard curve (0–1.0 mg mL⁻¹) and expressed as milligrams of gallic acid equivalent per gram of dry weight (mg GAE g⁻¹ DW).

***Quantification of total flavonols:*** Total flavonols were quantified following the method described by Kim (2003). Each reaction consisted of mixing 135 μL of extract, previously diluted in water (230 μL of water and 40 μL of extract), with 7.5 μL of 5% NaNO₂. The mixture was vortexed and incubated for 5 min, followed by the addition of 30 μL of 2.5% AlCl₃. After homogenization and a 6-min incubation, 50 μL of 1 M NaOH and 50 μL of distilled water were added. After 5 min, the absorbance was measured at 510 nm against a water blank. The total flavonol concentration was calculated based on a quercetin standard curve (0–1.0 mg mL⁻¹) and expressed as milligrams of quercetin equivalent per gram of dry weight (mg EQ g⁻¹ DW).
